# Modification of the Nutritional Quality and Oxidative Stability of Lupin (*Lupinus mutabilis* Sweet) and Sacha Inchi (*Plukenetia volubilis* L.) Oil Blends

**DOI:** 10.3390/molecules27217315

**Published:** 2022-10-27

**Authors:** Gilbert Rodríguez, Elza Aguirre, Any Córdova-Chang, Jenny C. Muñoz-Saenz, Mery Baquerizo, Andrea Brandolini, Eudes Villanueva, Alyssa Hidalgo

**Affiliations:** 1 Escuela de Ingeniería Agroindustrial, Universidad Nacional del Santa, Chimbote, Ancash 02712, Peru; 2 Facultad de Medicina Humana, Universidad Peruana los Andes, Huancayo 12006, Peru; 3 Facultad de Ciencias Aplicadas, Universidad Nacional del Centro, Av Mariscal Castilla 3909, Huancayo 12006, Peru; 4 Consiglio per la Ricerca in Agricoltura e L’analisi Dell’economia Agraria—Centro di Ricerca Zootecnia e Acquacoltura (CREA-ZA), Viale Piacenza 29, 26900 Lodi, Italy; 5 Departamento Académico de Ingeniería en Industrias Alimentarias e Ingeniería Forestal y Ambiental, Universidad Nacional Autónoma de Tayacaja Daniel Hernández Morillo, Huancavelica 09156, Peru; 6 Department of Food, Environmental and Nutritional Sciences (DeFENS), Università degli Studi di Milano, via Celoria 2, 20133 Milan, Italy

**Keywords:** fatty acids, Rancimat, shelf-life, thermodynamics values, tocopherols, ω3/ω6 ratio

## Abstract

Andean lupin (*Lupinus mutabilis*) oil is rich in monounsaturated (54.2%) and polyunsaturated (28.5%) fatty acids but has a ω-3:ω-6 ratio (1:9.2) above the recommended values for human health. Sacha inchi (*Plukenetia volubilis*) oil presents a high polyunsaturated fatty acid content (linolenic 47.2% and linoleic 34.7%), along a ω-3:ω-6 ratio (1:0.74) good for human consumption. The objective of this research was to study the physico-chemical properties and oxidative stability of tarwi and sacha inchi oil blends (1:4, 1:3, 1:1, 3:1 and 4:1 *w*:*w*) with suitable ω-3:ω-6 ratios. All blends showed ω-3:ω-6 ratios between 1:0.8 and 1:1.9, acceptable from a nutritional point of view, and high total tocopherols’ content (1834–688 mg/kg), thanks to sacha inchi. The oxidative stability index (OSI) of the mixtures by the Rancimat method at 120 °C ranged from 0.46 to 8.80 h. The shelf-life of 1:1 tarwi/sacha inchi oil blend was 1.26 years; its entropy (−17.43 J/mol), enthalpy (107.04 kJ/mol), activation energy (110.24 kJ/mol) and Gibbs energy (113.76 kJ/mol) suggest low oxidation reaction rates and good stability. Hence, balanced blends of tarwi/sacha inchi oils can achieve optimal nutritional properties and enhanced shelf-life.

## 1. Introduction

The technological application and commercialization of vegetable oils are determined by their physical and chemical properties; however, their nature can be modified by different methods to better suit specific needs and health concerns. For example, hydrogenation improves oxidative stability and texture, albeit may convert some *cis* fatty acids double-bonds to *trans*, leading to a negative impact on human health [1]. Interesterification does not generate isomerization or saturation of fatty acids, but requires expensive equipment [2,3]. Fractioning takes advantage of melting point and texture properties, but usually is only a step before hydrogenation, interesterification or oils mixing [4]. On the other hand, oil mixing is a simple and widely employed method that allows the successful exploitation of the properties of different oils [5]. For example, chia and sesame oils are mixed to improve the ω-3:ω-6 ratio and to increase the shelf life [6]. The 80:20 canola: olive oil blend, after being mixed with 20% palm olein, showed good cold stability test and low oxidation during deep-frying [7]. Supplementing soybean oil with 20% sea buckthorn oil, camellia oil, rice bran oil, sesame oil or peanut oil increased the monounsaturated fatty acids and tocopherol content, leading to an improved oxidative stability [8]. The addition of up to 20% rice bran oil to rapeseed oil increased tocotrienols, β-sitosterol and squalene, while a similar addition of black cumin seed oil augmented the content of α- and γ-tocopherols as well as of all tocotrienol homologues; in general, the blends lowered the polyunsaturated/saturated fatty acids ratios, raised the ω-3:ω-6 ratio and improved the oxidative stability [9]. Camellia oil, due to the high content of phenolic compounds and monounsaturated fatty acids, when added to soybean oil, produced blends with significantly improved frying stability [10].

Sacha inchi (*Plukenetia volubilis* L.) seeds have a high lipid content (33.4–54.3%), rich in polyunsaturated fatty acids (PUFA), α-linolenic acid (46–54%) and linoleic acid (33–37%) [11,12,13,14]. PUFAs present important benefits for health and nutrition because they contribute to the prevention of cardiovascular diseases; on the other hand, they decrease oxidative stability and reduce shelf life [10,11,12,13,14,15]. Andean lupin, also known as tarwi (*Lupinus mutabilis* Sweet), seeds contain 14–20% lipids [16], which are rich in monosaturated oleic acid (≈56%) and polyunsaturated linoleic acid (18.4–26.1%) [17,18].

A diet with a balanced ω-3:ω-6 ratio affords numerous health benefits, because these PUFAs are precursors of eicosanoids (prostaglandins, thromboxanes, leukotrienes) that intervene in many physiological processes such as blood clotting or inflammatory and immunological responses [19,20]. In fact, the low ω-3:ω-6 ratios of canola (1:5.6) and fish (2:1) oils prevent the development of atherosclerosis and macrophage-induced foam cell formation, while the high ω-3:ω-6 ratios of corn (52:1) and olive (13.4:1) oils have the opposite effects [21]. The ω-3:ω-6 ratio may also serve as a biomarker in the treatment of type 2 diabetes mellitus [22]. Consumption of mixtures of canola oil, walnut oil, sesame oil and wheat germ oil (1:1:1:1 ratio) may be useful against kidney diseases due to their anti-diabetic and antioxidant properties [23]. Therefore, vegetable oil blends with a carefully balanced composition can achieve increased nutritional value, longer shelf life and greater economic benefits [5,23,24,25].

The objective of this research was to determine the physico-chemical characteristics, oxidative stability, activation energy (Ea), enthalpy (ΔH^++^), entropy (ΔS^++^), Gibbs free energy (ΔG^++^), Q_10_ and shelf life of different tarwi/sacha inchi oil blends, comparing them to pure tarwi and sacha inchi oils.

## 2. Results and Discussion

The extraction yield of the tarwi oil was 11.2 ± 0.8 g/100 g seed, while the lipid content in the cake was 9.8 ± 0.31 g/100 g; hence, the total lipids in the Andenes seeds were 21.0 g/100 g, leading to a 53% recovery, a value in the lower end of the range (50.0–75.7%) for expeller press extraction performed at 70 °C [26]. The sacha inchi yield was 38.5 g/100 g seed, with a lipid content in the cake of 12.1 g/100 g, for a total of 50.6 g/100 g seed and a 76% recovery.

### 2.1. Chemical Characterization

The fatty acid composition of the oils is reported in Table 1. Tarwi oil contained 54.2% oleic acid and 25.7% linoleic acid, while sacha inchi oil had 47.2% linolenic acid and 34.7% linoleic acid. Overall, the monounsaturated fatty acids (MUFA) content in tarwi oil was similar to the ≈50% observed in different *Lupinus mutabilis* varieties [26,27], while PUFA (28.5%) and saturated fatty acids (SFA) (17.3%) were the same as those reported for the same species [18]. On the other hand, sacha inchi oil showed low MUFA (9.4%) and high PUFA (82.0%), close to the published results [14,15]. The ω-3:ω-6 ratios were in the ranges reported for *Lupinus mutabilis* [18] and for sacha inchi [11]. The M1, M2 and M3 mixtures presented values between 1:0.8–1:0.9, close to the ω-3:ω-6 ratio of sacha inchi oil (1:0.7), and the M4 (1:1.2) and M5 (1:1.9) mixtures were significantly different from the values of sacha inchi and *Lupinus mutabilis* oils (*p* < 0.05). A ω-3:ω-6 ratio between 1:2 and 1:3 plays a fundamental role in the secondary prevention of chronic metabolic and immunomodulatory diseases such as colon cancer and rheumatoid arthritis [28].

In fact, taking into consideration homeostasis and normal human development during the life cycle, Simopoulos [29] noticed that today’s ω-3:ω-6 Western diet ratio (1:10–1:20) “is far from optimal and is highly inappropriate for normal growth and development” and suggested a ratio between 1:1 and 1:2 as the preferred target for human nutrition. There are no guidelines for the ω-3:ω-6 ratio, but current recommendations for the ω-3 and ω-6 intake can be used to calculate a suggested dietary ratio, which may also vary by age and sex; however, ratios below 1:10 are generally considered acceptable (for a review, see [30]). All the samples presented peroxide value, *p*-anisidine value, acidity and total oxidation value (Table 1) within the acceptable ranges for vegetable oils [31], indicating good quality of the raw seeds, proper management of the extraction procedures and correct storage practices.

Tarwi oil had a much lower α-, γ- and δ-tocopherols content than sacha inchi oil (*p* ≤0.05). A similar profile, plus β-tocopherol, was reported for an Andenes lupin commercial sample extracted at higher temperature (70 °C) [26]. Overall, the total tocopherols concentration in tarwi oil was 294.4 mg/kg (Table 1), somewhat inferior to the 570 mg/kg of a similar oil [26], possibly because of a different cropping environment and the storage conditions of the seeds before extraction. The tocopherol content was lower than expected, considering the concentration found in debittered beans [16,32]. Nevertheless, the content depends not only on the ecotype, the environment and the year, but also on the extraction method and its conditions, which influence extraction rate and tocol rate: actually, the tocopherol analysis of lupin flours includes a saponification step (necessary to free esterified tocols) followed by an extraction with solvents [16]. Sacha inchi tocopherol content was akin to the result (2540 mg/kg) of an ecotype from the San Martín department, Peru [14], as well as to the value (2294 mg/kg) reported for a sample collected in the Loreto department, Peru [33]. In both species, γ-tocopherol, which displays the highest antioxidant activity [34], was the most abundant homologue, as also observed by other authors [15,16,33]. The different blends gave results closely reflecting the proportions between the two oils employed. Among high-PUFA oils, sacha inchi has more tocopherols than chia (718 mg/kg; [35]) and flaxseed (732–951 mg/kg; [36]). PUFA are highly susceptible to oxidation, but the presence of relevant concentrations of natural antioxidants, such as tocopherols, counters their degradation. In fact, a high oxidation stability of sacha inchi oil during frying was reported and was attributed to the preservation effect of tocopherols [14].

The carotenoid lutein, another well-known antioxidant molecule with beneficial health effects [37], was not detected in sacha inchi oil but was present, in a low quantity, in the blends (0.3–1.1 mg/kg) and in Andean lupin oil (1.4 mg/kg). This last value was inferior to that expected, considering the concentrations (0.68–3.25 mg/kg and 1.17–1.48 mg/kg DM) observed in tarwi seeds [16,32]; however, the same factors discussed above for tocol content also apply to this compound. The blends showed results in line with the proportions between the two oils employed.

### 2.2. Oxidative Stability Index

**Figure 1:**
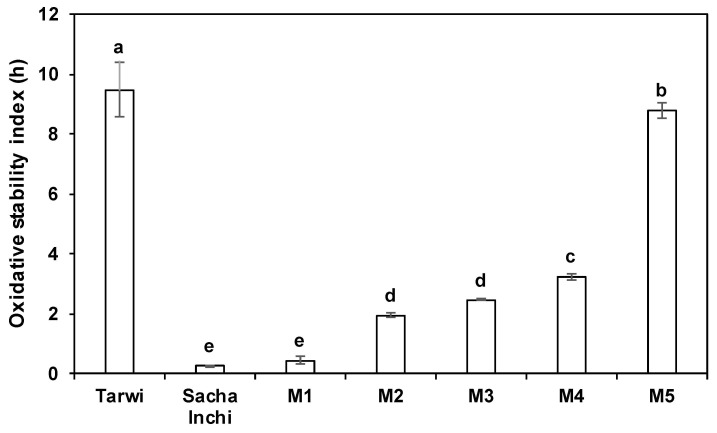
Oxidative stability index (OSI) at 120 °C of tarwi oil, sacha inchi oil and their blends. Tarwi oil: Sacha inchi oil ratios (*w*/*w*): M1 1:4; M2 1:3, M3 1:1; M4 3:1; M5 4:1. Different letters (a–e) indicate significant differences between samples at *p* ≤ 0.05 (*n* = 3).

In spite of the high tocols’ content, at 120 °C the OSI of the PUFA-rich sacha inchi oil was lower than that of the MUFA-rich tarwi oil. Hence, the growing addition of tarwi oil in the blends increased their OSI from 0.46 (M1) to 8.80 h (M5) (Figure 1). The OSI values at different temperatures (from 80 to 140 °C) for the controls and the M3 blend (selected a priori as a good balance between the two oils) are presented in Table 2.

The tarwi oil results were not far from those (2.50 and 9.97 h at 140 °C and 120 °C, respectively) reported by Salvatierra-Pajuelo et al. [38], but were lower than those (2.6, 13.9 and 29.0 h at 140, 120 and 110 °C, respectively) noticed by Pascual-Chagman et al. [26], a difference coherent with the superior tocopherol content described above. The sacha inchi oil scores were similar to those (0.49 h at 110 °C, 1.59 h at 100 °C, 4.64 h at 90 °C and 20.5 h at 90 °C) observed by Rodríguez et al. [39]. Nevertheless, at 100 °C the OSI was lower than that (4.32 h) described by Rodríguez et al. [14], possibly because of year/season influence on fatty acids composition, as suggested by the superior SFA (8.7% vs. 5.2%) and MUFA (9.4% vs. 7.6%) contents reported.

The rate of auto-oxidation more than doubled for each 10 °C temperature increase; thus, the OSI values augmented sharply as the temperature decreased from 140 to 80 °C. The relevant concentrations of unsaturated fatty acids in the oils played a fundamental role in their oxidative instability, because lower OSI are correlated to high degrees of unsaturation [40]. In fact, high concentrations of unsaturated fatty acids reduce OSI, while an increase in SFA and MUFA content augments OSI [40,41]. However, the high Rancimat test temperatures influence the induction times because the polymerization releases volatile compounds and may cause the formation of a dry film that could limit oxygen access to the samples [42], thus leading to a significant underestimation of the real shelf life [43].

### 2.3. Thermodynamic Study and Shelf Life

The ΔH^++^ and ΔS^++^ values (Table 3) showed significant differences (*p* ≤ 0.05) among treatments. The positive activation enthalpies (ΔH^++^ > 0) and negative entropies (ΔS^++^ ≤ 0) suggest an endothermic nature of the activated complex formation [44]. During the auto-oxidation, tarwi oil absorbed less heat than sacha inchi and M3 oils, an unexpected result since its OSI was the highest at 110–120 °C. The negative entropies suggest that the activated complexes were more ordered than their reactants; additionally, high negative values (such as in sacha inchi) imply that fewer species are involved in the activated complex state, which will have a lower potential and therefore a slower oxidation reaction rate. These results determined positive Gibbs energy values (ΔG^++^ > 0), suggesting that at these temperatures the auto-oxidation process is not spontaneous; sacha inchi oil had a higher ΔG^++^ value than the other samples, thus showing lower oxidation reaction rates.

The activation energy (Ea) in the autoxidation process of oils denotes a delay in the initial oxidation process due to the rupture of the fatty acid chains and is influenced by the unsaturation levels. Hence, a high content of linoleic and/or linolenic acids should decrease the Ea, while a high oleic acid and saturated fatty acids percentage should increase it [24]. However, sacha inchi oil presented an Ea value (111.6 kJ/mol) marginally higher than tarwi oil (108.2 kJ/mol), despite its superior PUFA content; an explanation for this phenomenon may be the antioxidant influence of its tocopherols. Rodríguez et al. [39] reported a slightly superior Ea (137.9 kJ/mol) for sacha inchi using the same Rancimat method, while lower activation energies were described for chia oil (82 kJ/mol [6,45] and for sesame oil (96–99 kJ/mol) [6,46]. The Ea of the M3 blends fell within those of the two pure oils, leading to an oxidation faster than the Andean lupin oil and a stability higher than the sacha inchi oil.

Table 4 shows the linear relationship (R^2^: 0.97–1.00) between temperature and Log(OSI) for the tarwi, sacha inchi and M3 blend oils, with α values that range between −0.036 and −0.051. The shelf life of tarwi, M3 and sacha inchi oils at 25 °C was 2.57, 1.26 and 1.11 years, respectively. The sacha inchi shelf life was lower than the 1.79 years related by Rodríguez et al. [39] for sacha inchi oils stored at 25 °C but was much higher than the 25 days for chia oil, despite similar PUFA contents [6]. The M3 mixture increased the shelf life by 0.15 years compared to pure sacha inchi oil. The Q_10_ values varied between 2.26 (tarwi) and 2.62 (sacha inchi), i.e., were slightly superior to those of other vegetable oils such as chia (2.03), sesame (2.12), soybean (1.99–2.09), corn (2.01) and canola (2.01) [6,44]. Hence, the higher Q10 of our oils confirms their longer shelf life compared with these other vegetable oils.

## 3. Materials and Methods

### 3.1. Materials

*Lupinus mutabilis* seeds of the variety Andenes from the Ancash region (latitude: 12°04′55″ S, longitude: 76°56′53″ W, altitude 878 m a.s.l), Peru, were obtained from the Programa de Leguminosas of the Universidad Nacional Agraria La Molina (Lima, Peru). After removing the bitter and toxic quinolizidine alkaloids by repeated washing [47], they were dried to 10.5 ± 0.10 g/100 g moisture. Sacha inchi seeds were collected in Lamas, San Martin region (latitude: 06°25′19″ S, longitude: 76°30′58″ W, altitude 808 m a.s.l), Peru and were dried to 9.1 ± 0.12 g/100 g moisture. The drying process of the seeds was carried out in a SW-17TC-1 oven (Blue M, New Columbia, PA, USA).

### 3.2. Oils Extraction and Blends Preparation

The tarwi and the sacha inchi oils were extracted from the seeds by cold pressing (max temperature 50 °C), using a single-screw CA 59 G Komet expeller (IBG Monforts Oekotec, Mönchengladbach, Germany) at a screw speed of 35–40 rpm, clarified by centrifugation and stored at 4.0 ± 0.5 °C in dark flasks under nitrogen atmosphere. Five tarwi/sacha inchi oil blends, M1 (1:4 *w*:*w*), M2 (1:3), M3 (1:1), M4 (3:1) and M5 (4:1) were prepared and thoroughly mixed for 30 s with a vortex (Velp Scientifica, Usmate Velate, Italy).

### 3.3. Chemical Analyses

The fatty acids’ (FA) composition was determined as fatty acid methyl esters by gas chromatography after transesterification of the oils with 2 N KOH in methanol, according to Standard Method 2.302 [48]. The chromatographic analysis was performed with a GC-2010 gas chromatograph (Shimadzu, Kyoto, Japan) including a flame ionization detector and an AOC-20Si autosampler (Shimadzu, Kyoto, Japan). The capillary column was a SP^®^-2560 (100 m × 0.25 mm, df 0.2 μm, Restek, Bellefonte, PA, USA). The operative conditions were: carrier He at 261.5 kPa and at 30 mL/min; oven temperature 100 °C for 4 min, increased by 3 °C/min to 240 °C, kept at 240 °C for 10 min; injection temperature 225 °C; flame ionization detector temperature 250 °C. The injection volume was 1 µL. Monounsaturated fatty acids (MUFA)/polyunsaturated fatty acids (PUFA), PUFA/saturated fatty acid (SFA) and ω-3:ω-6 ratios were computed from the fatty acids’ data.

Peroxide value (PV), index of primary oxidation, was determined according to the method Cd 8-53 [49] and expressed as milliequivalents of active oxygen per kilogram of oil (meq active oxygen/kg oil), *p*-anisidine value (*p*-AV), index of secondary oxidation, was analyzed following the method Cd 18-90 [49] and acidity value (mg KOH/g oil), index of lipid hydrolysis, was performed according to method Cd 3d-63 [49], while total oxidation value (ToTox) was computed as 2PV + *p*-AV [50].

The tocols were determined according to Rodríguez et al. [14]. Briefly: solutions in hexane: isopropyl alcohol (99.0:1.0 *v*/*v*) at concentrations of 10 mg/mL and 100 mg/mL were filtered through a 0.2 µm PTFE and immediately analyzed by NP-HPLC under the following system and operating conditions: Adamas^®^ Silica column 250 × 4.6 mm, 5 μm, and guard cartridge 10 × 4.6 mm, 5 μm (Sepachrom SRL, Rho, Italy); mobile phase, hexane: ethyl acetate: acetic acid (97.3:1.8:0.9, *v*/*v*/*v*); flow rate, 1.6 mL/min; pump L-2130 Elite LaChrom (VWR, Hitachi, Japan); fluorimetric detector Jasco 821 FP Intelligent Spectrofluorometer (Japan) at excitation–emission wavelengths of 290 nm and 330 nm, respectively; connected to a computer with the software Empower 2 (Waters Chromatography Division, Millipore, Milford) through the Waters e-SAT/IN module. The tocols’ standard curves were built using eleven concentrations (between 0.40 and 109.73 mg/L) of α-tocopherol standard (Fluka, St. Louis, MO, USA), sixteen concentrations (between 0.20 and 23.20 mg/L) of γ-tocopherol standard (Supelco, Bellefonte, PA, USA) and eleven concentrations (between 0.05 and 9.35 mg/L) of δ-tocopherol standard (Supelco, Bellefonte, PA, USA). The total tocopherols were computed as the sum of the different homologues.

The carotenoids were assessed as outlined by Varas Condori et al. [43] on the oil solutions in hexane: isopropyl alcohol (90:10 *v*/*v*) at a concentration of 100 mg/mL. The mixes were filtered through a 0.2 μm PTFE filter, and analyzed immediately by NP-HPLC. The following system and operating conditions were used: Adamas^®^ Silica column, 250 × 4.6 mm, 5 μm and guard cartridge 10 × 4.6 mm, 5 μm (Sepachrom SRL, Rho, Italy); column oven at 20 °C L-2300 Elite LaChrom (VWR, Hitachi, Japan); mobile phase, hexane: isopropyl alcohol (5%); flow rate, 1.5 mL/min; pump L-2130 Elite LaChrom (VWR, Hitachi, Japan). The carotenoids were detected at 450 nm by Diode Array Detector L2450 Elite LaChrom (Merck, Hitachi, Japan) set in the range of 200–650 nm. The HPLC system was controlled by the software EZChrom Client/Server version 3.1.7. For peak quantification, a calibration curve (between 0.3 and 3.0 mg/L) of the lutein standard (Fluka, St. Louis, MO, USA) stock solution was used. The tocols and the carotenoids are reported as mg/kg oil. 

### 3.4. Oxidative Stability Index (OSI)

The OSI (in hours) of the two oils and of their blends was evaluated according to method Cd 12b-92 [49] using a 743 Rancimat equipment (Metrohm Schweiz AG, Zofigen, Switzerland). The assays were carried out at 80, 90, 100, 110, 120, 130 and 140 °C using 3.0 ± 0.1 g of oil with an air flow of 15 L/h. The temperatures were carefully chosen according to the nature of the oils and their resistance to oxidation: if the temperature is too high, the analysis time is very short, but if the temperature is too low, then the analysis time is exceedingly protracted [35,39,45].

#### Thermodynamic Analysis and Shelf Life

The activation energy (Ea) was determined from the slope of the line representing the natural logarithm of the OSI values vs. the inverse of the absolute temperature (1/T) [46]:

(1) $$\mathrm{Ln}\left(\mathrm{OSI}\right)=\mathrm{Ln}\left(\frac{-\mathrm{Ln}\left(1-\alpha^{*}\right)}{Z}\right)+\frac{E_{a}}{\mathrm{RT}}$$

where α* is the level of transformation of unsaturated molecules; Z is the Arrhenius equation factor and R is the universal gas constant (8.314 J/mol).

The activation enthalpy (ΔH^++^) and entropy (ΔS^++^) were determined as regression of the K/T logarithm vs. 1/T [41]:

(2) $$\mathrm{Log}\left(\frac{K}{T}\right)=\mathrm{Log}\left(\frac{k_{B}}{h}\right)+\left(\frac{∆S^{++}}{2.303R}\right)-\left(\frac{∆H^{++}}{\mathrm{RT}}\right)$$

where K is the inverse of OSI; T is the absolute temperature; h is Planck’s constant (6.6260755 × 10^−24^ J s); k_B_ is Boltzmann’s constant (1.380658 × 10^−23^ J/K) and R is the universal gas constant (8.314 J/mol). The Gibbs free energy (ΔG^++^) was computed as ΔG^++^ = ΔH^++^ − TΔS^++^. The shelf life was determined by extrapolating the linear correlation of the OSI logarithm at T = 25 °C [41]: Log (OSI) = α(T) + β. The Q_10_ temperature coefficient, which indicates the increase in reaction rate after a 10 °C rise in temperature, was obtained from the (OSI at T)/(OSI at T + 10 °C) [51].

### 3.5. Statistical Analysis

The analyses were carried out on three independent samples (tocols and carotenoids in double on each sample) and the data underwent one-way ANOVA; when differences were found at *p* ≤ 0.05, Fisher’s LSD at 95% significance level was computed. All analyses were performed using Statgraphics^®^ Centurion XVI (Statpoint Technologies, Inc., Warrenton, Virginia, USA). Average values and standard deviations (SD) were computed with the Excel^®^ program (Microsoft, Redmond, WA, USA).

## 4. Conclusions

Tarwi and sacha inchi oils are rich in monounsaturated and polyunsaturated fatty acids, respectively, while their blends show a more balanced ω-3:ω-6 compositions, nutritionally preferable for human consumption and potentially preventative against diseases such as rheumatoid arthritis and colon cancer. All the blends present very high tocopherol concentrations, low PV and acidity indices, mainly due to the sacha inchi oil. On the other hand, the oxidative stability of the blends improved with increasing the tarwi oil content. In particular, the 1:1 blend studied reached a shelf life of 1.26 years, very appealing for commercial development. In conclusion, balanced blends of tarwi/sacha inchi oils can achieve optimal nutritional properties along with enhanced shelf-life.

## Figures and Tables

**Table 1:** Mean (± standard deviation) fatty acid composition (%), peroxide value (PV; meq active oxygen/kg oil); *p*-anisidine value (*p*-AV); acidity (mg KOH/g oil); total oxidation value (ToTox = 2PV + p-AV); tocopherol and carotenoid content (mg/kg) of tarwi oil, sacha inchi oil, and their blends. Tarwi oil: Sacha inchi oil ratios (*w*:*w*) were 1:4 (M1), 1:3 (M2), 1:1 (M3), 3:1 (M4) and 4:1 (M5).

	Tarwi	Sacha Inchi	Blends				
			M1	M2	M3	M4	M5
C16:0	10.9 ± 0.01 ^a^	4.8 ± 0.09 ^g^	5.2 ± 0.02 ^f^	6.1 ± 0.10 ^e^	6.8 ± 0.01 ^d^	8.8 ± 0.06 ^c^	10.2 ± 0.07 ^b^
C18:0	6.3 ± 0.27 ^a^	3.9 ± 0.04 ^g^	4.0 ± 0.01 ^f^	4.1 ± 0.04 ^e^	4.5 ± 0.08 ^d^	5.1 ± 0.09 ^c^	5.5 ± 0.09 ^b^
C18:1 (ω-9)	54.2 ± 1.03 ^a^	9.4 ± 0.03 ^g^	14.8 ± 0.01 ^f^	19.4 ± 0.05 ^e^	27.4 ± 0.13 ^d^	35.9 ± 0.86 ^c^	42.9 ± 0.10 ^b^
C18:2 (ω-6)	25.7 ± 0.50 ^d^	34.7 ± 0.52 ^a^	33.0 ± 0.02 ^a,b^	30.6 ± 0.34 ^b^	28.2 ± 0.62 ^c^	27.5 ± 0.09 ^c^	26.2 ± 0.01 ^d^
C18:3 (ω-3)	2.8 ± 0.45 ^g^	47.2 ± 0.59 ^a^	43.0 ± 0.04 ^b^	39.8 ± 0.15 ^c^	33.1 ± 0.65 ^d^	22.7 ± 1.10 ^e^	13.7 ± 0.03 ^f^
MUFA/PUFA	1.90 ± 0.001 ^a^	0.11 ± 0.02 ^g^	0.19 ± 0.001 ^f^	0.28 ± 0.001 ^e^	0.45 ± 0.002 ^d^	0.72 ± 0.003 ^c^	1.07 ± 0.002 ^b^
PUFA/SFA	1.7 ± 0.01 ^g^	9.5 ± 0.10 ^a^	8.3 ± 0.05 ^b^	6.9 ± 0.01 ^c^	5.4 ± 0.05 ^d^	3.6 ± 0.11 ^e^	2.5 ± 0.01 ^f^
ω-3/ω-6	1:9.14 ^f^	1:0.7 ^a^	1:0.8 ^b^	1:0.8 ^b^	1:0.9 ^c^	1:1.2 ^d^	1:1.9 ^e^
PV	1.8 ± 0.01 ^e^	2.0 ± 0.02 ^a^	2.0 ± 0.02 ^b^	1.9 ± 0.02 ^c^	1.9 ± 0.01 ^c^	1.9 ± 0.01 ^c,d^	1.9 ± 0.01 ^d,e^
*p*-AV	1.1 ± 0.01 ^g^	1.4 ± 0.01 ^a^	1.4 ± 0.02 ^b^	1.3 ± 0.02 ^c^	1.3 ± 0.01 ^d^	1.2 ± 0.02 ^e^	1.2 ± 0.01 ^f^
Acidity	0.9 ± 0.01	1.1 ± 0.03	1.0 ± 0.01	1.0 ± 0.01	1.0 ± 0.02	1.0 ± 0.02	0.9 ± 0.01
ToTox	4.8 ± 0.02 ^e^	5.5 ± 0.03 ^a^	5.3 ± 0.05 ^b^	5.1 ± 0.04 ^c^	5.1 ± 0.02 ^c^	5.0 ± 0.03 ^d^	4.9 ± 0.02 ^d^
α–tocopherol	1.60 ± 0.13 ^c^	3.26 ± 0.19 ^a^	3.08 ± 0.21 ^a^	2.77 ± 0.07 ^a^	2.21 ± 0.27 ^b^	2.12 ± 0.13 ^b,c^	2.08 ± 0.28 ^b,c^
γ-tocopherol	288.2 ± 1.0 ^f^	1296.5 ± 94.2 ^a^	1157.7 ± 16.2 ^b^	998.7 ± 32.3 ^c^	817.5 ± 35.2 ^d^	663.6 ± 74.8 ^e^	511.6 ± 2.9 ^e^
δ-tocopherol	4.55 ± 0.11 ^g^	794.4 ± 45.5 ^a^	673.3 ± 1.6 ^b^	544.3 ± 44.8 ^c^	412.8 ± 15.5 ^d^	283.0 ± 51.2 ^e^	174.4 ± 3.6 ^f^
Total tocopherol	294.4 ± 0.7 ^g^	2094.1 ± 139.5 ^a^	1834.0 ± 17.6 ^b^	1545.8 ± 77.2 ^c^	1232.5 ± 51.0 ^d^	948.7 ± 126.1 ^e^	688.1 ± 1.0 ^f^
Lutein	1.44 ± 0.03 ^a^	nd ^e^	0.29 ± 0.02 ^c,d^	0.47 ± 0.05 ^c^	0.86 ± 0.12 ^b^	0.94 ± 0.13 ^b^	1.10 ± 0.29 ^b^

n.d.: not detected, lower than the detection limit. Different letters (a–g) in the same row indicate significant differences at *p* ≤ 0.05 among samples (*n* = 3). SFA: Saturated fatty acids; MUFA: monounsaturated fatty acids; PUFA: polyunsaturated fatty acids.

**Table 2:** Mean (± standard deviation) oxidative stability index (h) of tarwi oil, sacha inchi oil and their 1:1 blend (M3).

Temperature (°C)	Tarwi	Sacha Inchi	M3
80		17.48 ± 0.45 ^a^	
90		3.54 ± 0.12 ^b^	
100		1.57 ± 0.08 ^c^	18.28 ± 0.06 ^a^
110	19.84 ± 0.01 ^a^	0.50 ± 0.01 ^d^	4.70 ± 0.19 ^b^
120	9.48 ± 0.91 ^b^	0.20 ± 0.01 ^d^	2.46 ± 0.02 ^c^
130	3.58 ± 0.08 ^c^		1.21 ± 0.03 ^d^
140	1.76 ± 0.03 ^d^		

Different letters (a–d) in the same column indicate significant differences between samples at *p* ≤ 0.05 (*n* = 3).

**Table 3:** Thermodynamics values (mean ± SD) for enthalpy (∆H^++^), entropy (∆S^++^), Gibbs free energy (∆G^++^) and activation energy (Ea) of tarwi oil, sacha inchi oil and their 1:1 blend (M3).

	Tarwi	Sacha Inchi	M3
∆H^++^ (kJ/mol)	104.95 ± 1.07 ^b^	112.01 ± 1.65 ^a^	107.04 ± 0.27 ^b^
∆S^++^ (J/mol)	−1.84 ± 2.54 ^a^	−50.85 ± 4.26 ^c^	−17.43± 0.79 ^b^
∆G^++^ (kJ/mol)	105.65 ± 2.03 ^c^	131.47 ± 3.13 ^a^	113.77 ± 0.47 ^b^
Ea (kJ/mol)	108.24 ± 1.07 ^b^	115.13 ± 1.64 ^a^	110.24 ± 0.27 ^b^

Different letters (a–c) in the same row indicate significant differences between samples at *p* ≤ 0.05 (*n* = 3).

**Table 4:** Mean (± SD) shelf life (oxidative stability index at 25 °C in years; OSI25) and Q10 (increase in reaction rate due to a 10 °C temperature rise) of tarwi oil, sacha inchi oil and their 1:1 blend (M3).

	Log OSI = α(T) + β			OSI_25_	Q_10_
	α	β	R^2^		
Tarwi	−0.036 ± 0.000	5.237 ± 0.065	0.995	2.57 ± 0.32 ^a^	2.26 ± 0.35 ^a^
Sacha inchi	−0.051 ± 0.001	5.253 ± 0.047	0.980	1.11 ± 0.08 ^b^	2.62 ± 0.43 ^a^
M3	−0.038 ± 0.000	4.992 ± 0.007	0.966	1.26 ± 0.01 ^b^	2.61 ± 0.91 ^a^

α and β: constants; R^2^: coefficient of determination. Different letters (a,b) in the same column indicate significant difference between samples at *p* ≤ 0.05 (*n* = 3 for OSI_25_, *n* = 9 for Q_10_).

## Data Availability

All the data related to this work are given here in the manuscript.
